# Unveiling the Link Between Breast Cancer Treatment and Osteoporosis: Implications for Anticancer Therapy and Bone Health

**DOI:** 10.1155/2024/5594542

**Published:** 2024-11-14

**Authors:** Bhawna Goel, Tarun Virmani, Vikas Jain, Girish Kumar, Ashwani Sharma, Abdullah Al Noman

**Affiliations:** ^1^School of Pharmaceutical Sciences, MVN University 121102, Palwal, Haryana, India; ^2^Amity Institute of Pharmacy, Amity University, Greater Noida 2011308, Uttar Pradesh, India; ^3^Department of Pharmacy, JSS College of Pharmacy, Sri Shivarathreeshwara Nagara 570015, Mysuru, Karnataka, India; ^4^Department of Pharmaceutics, Delhi Pharmaceutical Sciences Research University, Delhi, India; ^5^School of Pharmacy, BRAC University, Dhaka, Bangladesh

**Keywords:** body mineral density (BMD), breast cancer, correlation, medication, osteoporosis

## Abstract

**Background:** The interplay between breast cancer treatment and osteoporosis has important consequences for anticancer therapy and patient bone health. Many breast cancer therapies involve hormonal treatments that lower estrogen levels, which can lead to an increased risk of osteoporosis due to reduced bone mineral density. Aromatase inhibitors, chemotherapy, and surgeries such as oophorectomy can further aggravate bone loss, highlighting the necessity of prioritizing bone health during cancer treatment.

**Objective:** This review is aimed at investigating the complex relationship between breast cancer therapies and bone health by examining the molecular and cellular mechanisms through which anticancer treatments lead to bone loss. It also seeks to assess the effects of various treatment options, such as selective estrogen receptor modulators (SERMs) and bisphosphonates, on reducing bone loss and maintaining bone health during cancer therapy.

**Method:** The review explores the mechanisms underlying bone loss in breast cancer patients undergoing treatment, focusing on factors such as estrogen depletion, inflammatory cytokines, and changes in bone remodelling processes. Additionally, it evaluates the efficacy of different therapeutic interventions, including pharmacological treatments like bisphosphonates and third-generation SERMs, in mitigating bone-related side effects.

**Results:** The findings indicate a critical need to balance the effectiveness of breast cancer treatments with the preservation of bone health. Pharmacological treatments like bisphosphonates and denosumab have been identified as essential for managing bone health in breast cancer patients. Furthermore, third-generation SERMs show potential in reducing bone loss associated with cancer therapy.

## 1. Introduction

Breast cancer (BC) is the most persistent malignancy and owns the second-largest death rate in women universally [1, 2]. Approximately 4.1 million women with a history of BC were calculated in 2022 with 339,250 active cases with 43,250 death cases [3]. BC begins in the breast tissue and, if untreated, has the potential to spread to other parts of the body [4]. It affects about one in eight women throughout their lifetime. According to the studies, BC patient goes through various psychological stages, transitions, transformations, and exploration in surviving and diagnosing the disease [5]. One of the major transformations is situated with the bone microarchitecture which is characterized by severe pain, reduction in bone mineral density (BMD), impaired mobility, bone breakability, and a proclivity to develop pathologic fractures, spinal cord compression, bone marrow aplasia, and hypercalcemia [6–9]. These conditions are the chief cause of morbidity which may result in osteopenia/osteoporosis (OST), bone malignancies (osteolytic and osteoblastic), and multiple myeloma (inside bone marrow) [10]. As per the cohort study, BC treatments can increase the risk of osteoporosis and fractures in patients, with postmenopausal women (generally aged 50 and above) being especially susceptible due to age-related decreases in bone density [11]. Additionally, younger patients who undergo premature menopause as a result of cancer treatments may also face a higher risk of fractures, though this occurs through different mechanisms [11]. An estimated 200 million people are affected by osteoporosis worldwide, and amongst them, 68% of people have a prevalence of BC-induced osteoporosis, and over 8.9 million osteoporosis-related fragility fractures of the hip, spine, and wrist are reported annually [12, 13]. Although BC and OST initially appear unconnected, there is evidence to support the possibility of a link between the two. During menopause due to decreased circulating hormones (estrogen), bone loss is distressing in women with a 3%–5% rise in 5 years, which is one of the few characteristics that demonstrate an association between BC and OST in postmenopausal women. Certain important therapies, such as chemotherapy (CT) and hormone therapy, cause a decline in bone density and a reduction in estrogen levels in BCs that have estrogen receptors (ERs) [14–16]. Additional reasons include vitamin D insufficiency and a significant lifestyle including smoking, drinking excessively, and being inactive, all of which raise the risk of BC-associated osteoporosis [17, 18]. Some genetic abnormalities also enhance the risk; for instance, mutations in the COL1A1 and COL1A2 genes are linked to an increased risk of osteoporosis, whereas mutations in the BRCA1 and BRCA2 genes are linked to an increased risk of BC [19, 20]. According to epidemiologic research, approximately 70% of whole patients dying of BC have evidence of metastatic bone disease [21].

The present state of knowledge on the morphological and molecular subtypes of BC is covered in this review. A deeper comprehension of the biological processes of BC underlying osteoporosis development, which is influenced by BC metastasis, could lead to more targeted therapeutic strategies that address both cancer progression and bone health simultaneously. Here, we analyze BC treatments, which are the vital cause of osteoporosis. The key to finding innovative anticancer therapy targets for heterogeneous BC that can avoid serious conditions like osteoporosis can be observed in this article.

## 2. Distribution of BC—Histological and Molecular Classification

BC is a diverse illness with a spectrum of different subtypes, each with distinct biological traits that influence how patients adapt to different therapeutic interventions and their clinical prognoses [22, 23]. Using a hierarchical clustering method, groundbreaking research examining the gene expression profiles of breast tumours has classified them into “intrinsic subtypes” or “molecular subtypes,” depending on how closely related their gene expression patterns are [24]. By identifying four molecular subtypes of BC—Luminal A, Luminal B, Human Epidermal Growth Factor Receptor 2 (HER2-enriched or c-ERBB2), and basal-like [25–27] gene expression profiling has fundamentally altered our understanding of the disease. These intrinsic subtypes differ significantly in incidence, response to therapy, disease progression, survival, and imaging characteristics. But based on “histological” and “molecular” characteristics, there is a diminutive alteration in cataloguing the subtypes. Breast tumours that express the ER and/or progesterone receptor (PR) are quoted to be “hormone receptor-positive” BCs, whereas those that do not express the ER, PR, or HER2 are referred to as “triple-negative breast cancers” (TNBC) which is well-thought-out to be the most antagonistic [1, 28–30]. In 2000, Perou et al. were the first to find four molecular subtypes, including luminal, HER2-enriched, basal-like, and normal breast-like, in their study of changes in the gene expression patterns of 8102 human genes from 40 distinct malignant human breast tissue samples [31]. Later, Prat and Perou, in their studies, subdivided luminal type into Luminal A and Luminal B [32]. Coherence analysis reinforced the identification of Luminal A, Luminal B, HER2-enriched, and basal-like as the four primary intrinsic subtypes of BC [33]. In addition, the fifth intrinsic subtype of BC, claudin-low BC, was identified in a combined investigation of human and rodent mammary cancers in 2007 [34, 35].

Wellings and Jensen [36] disproved the long-held belief that different histological types of BC would develop from different microanatomical structures of the normal breast by demonstrating that the vast majority of invasive BCs and their in situ precursors originate from the terminal duct-lobular unit regardless of the histological type. Microarray-based investigation of 11 unique varieties of BC has shown that each special type is made up of tumours that are more homogeneous than IDC-NSTs (invasive ductal carcinoma no special types) and invasive lobular carcinomas (ILCs) at the transcriptome level [29]. Except for apocrine carcinomas, hierarchical cluster analysis showed that each histological special type under study related to just one molecular subtype [37]. According to earlier research, adenoid cystic, medullary, and metaplastic carcinomas consistently presented a basal-like phenotype, while tubular, mucinous, and neuroendocrine carcinomas consistently demonstrated a luminal phenotype [1, 28, 38]. None of the tumours, however, belonged to the subtype that resembled a normal breast, and 6% of the rarer forms of BC had a molecular apocrine character (Table 1). According to the WHO (2012), breast malignancies fall into two major categories: carcinomas or sarcomas, depending on which cell origin is involved [37]. Breast tumours known as carcinomas develop from the cells that line the lobules and terminal ducts that produce milk in the breast, or the epithelial component of the breast. Breast stromal cells, such as myofibroblasts and blood vessel cells, are the source of sarcomas, a far more uncommon type of BC (1% of initial BC). The majority of breast malignancies are carcinomas [41]. Based on their degree of invasiveness about the primary tumour sites, the several forms of BC within the broad category of carcinomas are distinguished. “Noninvasive (or in situ),” “invasive,” and “metastatic breast” cancers are the three main categories of frequent BCs [1, 42, 43].

## 3. Noninvasive (or In Situ) BC

The term “intraductal carcinoma” also refers to ductal carcinoma in situ (DCIS). DCIS is a noninvasive or preinvasive BC that spreads through ducts and distorts ductal architecture [43]. Even though DCIS is not an invasive cancer, in situ carcinomas have a high potential to progress to an invasive cancer (unilateral); hence, it is crucial to treat the patient effectively and promptly if they want to avoid acquiring invasive cancer [44, 45].

## 4. Invasive or Infiltrating BC

Invasive breast tumours penetrate the stromal tissue surrounding the lobules and ducts with cancer cells that also spread outside of those structures. Two-thirds of women, or those over 55, are affected by this aggressive variant. Two subgroups of invasive BCs are recognised: invasive ductal carcinoma (IDC): about 80% of all BCs are IDCs, and the most prevalent kind of BC. It develops from DCIS with a fibrous response to produce a mass and metastasizes through lymphatics and blood. Several subtypes in the IDC classification include cribriform carcinoma of the breast, papillary carcinoma of the breast, mucinous carcinoma (MC) of the breast, and tubular carcinoma of the breast [44, 45]. ILC: ILC is the second most frequent type that ranges between 10% and 15% of all carcinomas. It develops from isolated tumour cells or in CDH 1 mutation with minimal fibrous response and metastasizes through viscera. ILC can distress women of any age but is more widespread in older women [46, 47]. Invasive subtypes collectively account for 90%–95% of all BC cases. Invasive ductal and lobular carcinomas with different pathologic characteristics as well as distinctive molecular and genetic abnormalities develop as single cells or in sheets. It is crucial to distinguish between lobular and ductal carcinomas since they may have various prognoses and available treatments that differ from one another [48].

## 5. Metastatic BC

Late-stage BC that has spread to other body organs are metastatic or stage IV malignancies. Metastatic BC can spread to distant areas such as the lung, liver, bone, and brain as well as to lymph nodes under the armpit [44, 46–48]. Micrometastases may still be present in the body even after the original tumour has been removed, allowing cancer to spread and come back. Clinically, individuals may be diagnosed with metastatic illness (or de novo metastatic BCs) at the time of their initial therapy, or they may experience metastases months or years later. Unfortunately, about 90%–95% of instances of BC are detected during their early stages, but 30% of these women develop a metastatic disease that is still incurable today [49].

## 6. Inflammatory Breast Cancer (IBC)

IBC, which contributes 1%–6% of all BCs, is a rare and aggressive kind [50]. Breast swelling resembling inflammation, skin that is purple or red, and pitting or thickening of the breast skin are major symptoms of IBC [43, 45, 46]. These symptoms are likely brought on by cancer cells obstructing lymphatic veins in the skin. Mammograms usually fail to detect IBC, which frequently does not present as a breast lump. IBCs are performing more proactively, expanding rapidly, and enlarging [51]. IBC is initially identified when the BC cells have already penetrated the skin locally [50, 51].

## 7. Paget's Disease of Breast

Paget's disease is a noninvasive epidermal BC, which affects the nipple-areolar complex and is frequently misdiagnosed as benign breast ailments [52]. Around 1%–3% of all BC cases are caused by this rare type of BC, which starts in the breast ducts and travels to the skin around the nipple before reaching the area around the areola [53]. The majority of the cells are positive for the HER2 protein, and about half of the cells exhibit ER+ and PR+ positivity. Usually, a tissue sample is required to detect cancer; occasionally, the diagnosis is corroborated using a mammogram, ultrasound, or MRI [52–54].

## 8. Papillary Carcinoma

Papillary carcinoma is yet another extremely unusual kind of BC, contributing to almost 0.5%–1% of cases overall [55, 56]. With or without invasion, papillary carcinoma cells are commonly arranged in cellular proliferations (finger-like projections) that surround fibrovascular centres [57]. The majority of papillary carcinomas are invasive and are managed similarly to IDCs. The prognosis of invasive papillary carcinoma is typically better than other types of invasive BC [58].

## 9. Phyllodes Tumours (PTs)/Carcinoma

PTs, a different uncommon BC, are fibroepithelial neoplasms (2.5%), which often form in the breast stromal cells and have a unique morphology resembling leaves [59, 60]. 0.3%–1% of all BCs are caused by these tumours, of which 25% are aggressive and 60% are benign. Li–Fraumeni syndrome sufferers and women under the age of 40 had a higher risk of developing PT [61].

## 10. Angiosarcoma of the Breast

Angiosarcomas, a rare and aggressive kind of tumour with epithelial cells that cover blood or lymphatic veins, make up less than 0.05% of malignant tumours. This lesion's invasive tendencies, poor prognosis, and lack of typical radiologic signals are all features of the condition [62, 63].

## 11. Medullary Breast Carcinoma (MBC)

Medullary carcinoma, a rare, peculiar, and exceptional form of invasive BC, has certain histopathological characteristics [64]. Approximately 3%–5% of all breast carcinomas are MCBs, which have lymphoplasmacytic infiltration, syncytial growth (less than 75%), Grade 2/3 nuclei, a low likelihood of metastasis, and a good prognosis [65]. Higher BRCA1/2 mutation rates and a larger absence of the ER, PR, and HER2 receptors are found in MCBs, which are more curable than high-grade infiltrative carcinomas [66].

## 12. MC

MC, a rare and unusual histological disease, is MC, also referred to as colloid carcinoma. Most mucus-producing cancer cells that cause MC, which accounts for around 4% of all invasive BC, leak mucus. MC has a better prognosis than a variety of ductal and lobular carcinoma types. Women who are pre- and postmenopausal are more likely to have MC [67, 68].

## 13. Tubular Carcinoma

Tubular carcinoma is a distinct, well-differentiated tumour that is a low-proliferative Grade 1 neoplasm. It has a favourable prognosis and a low rate of local recurrence. TC, a member of the Luminal A type with high levels of ERs, accounts for between 1% and 4% of all incidences of BC [69–71].

## 14. Molecular or Intrinsic BC

Gene expression studies have identified a variety of distinct BC subtypes, and these subtypes differ significantly in terms of prognosis and the therapeutic targets that are present in the cancer cells. As previously mentioned, Prat and Perou, Naderi et al., and Sørlie concluded that BC subtypes can be divided into four categories based on the immunohistochemistry expression of hormone receptors [32–34]. This was supported by the cohort analysis. Several gene clusters associated with the expression of the ER (the luminal cluster), the expression of the HER2, proliferation, and a distinct gene cluster known as the basal cluster are now included in the list of intrinsic genes that distinguish these subtypes.

## 15. Luminal A

Between 50% and 60% of BCs are of the Luminal A subtype. Typically, these tumours have good prognoses and distinct histological types such as tubular, invasive cribriform, mucinous, and lobular. Additionally, they frequently have low nuclear pleomorphism, poor mitotic activity, and low histological grade. Luminal A has higher ER and lower levels of proliferation-related genes [23, 72].

## 16. Luminal B

The Luminal A subtype makes up 50%–60% of all BCs. These tumours often have good outcomes and distinctive histological types like tubular, invasive cribriform, mucinous, and lobular. They frequently also exhibit low nuclear pleomorphism, low histological grade, low mitotic activity, and low nuclear activity. Luminal A has increased ER and decreased levels of proliferation-related genes [23, 72].

## 17. HER2-Positive

HER2 is a member of this family and is one of four membrane tyrosine kinases. The HER2 receptor is produced by the HER2 gene, a protooncogene located on chromosome 17q21. As a result of ligand attachment to their extracellular domains, HER proteins undergo dimerization and extracellular domain transphosphorylation. The family or homodimerization itself is activated when produced at very high levels.

## 18. TNBC

**Figure 1:**
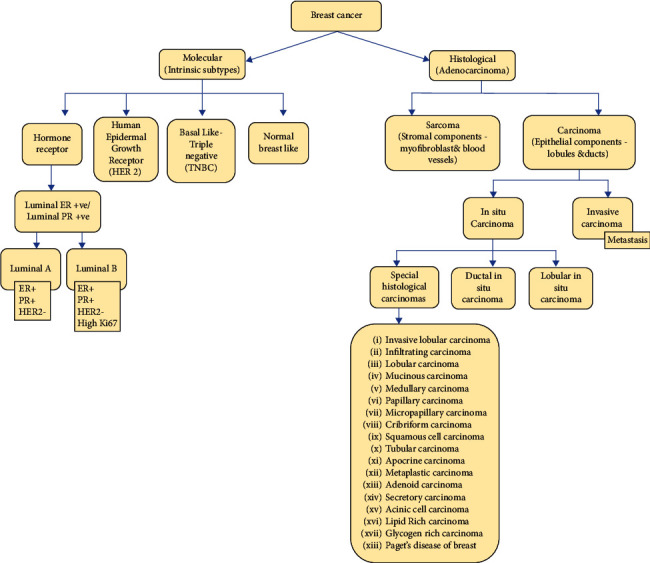
Image depicts different histological and molecular types of breast cancer.

TNBCs, having BRCA1/2 germline and PALB 2 mutations, express aberrant levels of PR, ER, and HER2 and are the most aggressive types [73]. This IDC made for 15%–20% of all incident BC, with a maximal 5-year survival rate and the worst prognosis [74]. Based on the results of gene expression profiling carried out on tumour samples from 587 TNBC patients, Lehmann et al. classified TNBC into six subgroups in 2011: basal-like 1 (BL1), basal-like 2 (BL2), mesenchymal (M), mesenchymal stem–like (MSL), immunomodulatory (IM), and luminal androgen receptor (LAR) [75, 76]. Additionally, they performed gene profiling on TNBC cell lines, analysed them, and split them into six subgroups, producing a solid cell model for clinical TNBC treatment [77, 78].  Figure 1 depicts different histological and molecular types of BC.

## 19. Metastasis of Different Types of BC to Bones

In its early stages, BC can harm the liver, lungs, brain, peritoneum, auxiliary lymph nodes, bones, and peritoneum. The most typical site of metastases in BC patients is the bone [79, 80]. Even after it has gone to the bones, cancer can typically still be treated to stop it from growing [81]. However, bone deterioration brought on by BC considerably worsens skeletal problems and lowers the quality of life. Up to 53.71% of BC patients with Stages I–III would develop bone disorders at 15 years of follow-up [82, 83]. BC metastasis to the bones has a high recurrence rate, with approximately 67% of BC tumours metastasizing to the bones. Breast tumours with Luminal B (79%) and Luminal A (70%), as opposed to HER2+ or TNBC (basal-like), are more likely to develop bone disease. Advanced BC will impact the liver and lungs in around 37% of cases, and the auxiliary lymph nodes in between 30% and 50% of cases [84]. It is uncertain how many instances had bone metastases [85]. The relative incidence of bone metastasis in individuals with advanced metastatic sickness varies by cancer type and is 65%–75% in BC, 65%–70% in the prostate, 60% in the thyroid, 30%–40% in the lung, 40% in the bladder, 20%–25% in renal cell carcinoma, and 14%–45% in melanoma. In different types of carcinomas, the median time to death after the diagnosis of bone metastasis is 6 months in the case of melanomas, 6–7 months for lung cancer, 6–9 months for bladder cancer, 12 months for renal cell carcinoma, 12–53 months for prostate cancer, 19–25 months for BC, and 48 months for thyroid cancer [84, 86–88]. Excruciating pain, limited mobility, pathologic fractures, spinal cord compression, and bone metastases are all symptoms of bone metastases, which are the primary cause of morbidity [85, 89].

## 20. Bone Remodelling

The lifelong process of bone remodelling maintains a balance between bone formation (osteogenesis) by osteoblasts (OBs) and resorption by osteoclasts (OCs), resulting in a mature, dynamic bone structure to safeguard internal organs [90, 91]. Remodelling also happens when a fractured bone is reshaped or when a small crack is repaired [92]. Progenitor stem cells which are housed by the periosteum (bone surface layer) are responsible for the production of “OBs” and “chondroblasts” [93].

### 20.1. OBs or Bone Formation

In nontumour-containing bone, remodelling begins when OBs sense microcracks or when bones are bearing much weight. At the site of resorption lacunae, a team of OBs produces an extracellular matrix containing Type 1 collagen and various noncollagenous proteins, such as osteopontin, osteocalcin, and osteonectin [93]. Vitamin D, calcium, and alkaline phosphatase help this matrix mineralize. The OB activates M-CSF (macrophage colony-stimulating factor), which helps to stimulate myeloid cells like monocytes [95, 96]. OB also produces a substance called RANKL (receptor activator of nuclear factor *κ*B ligand) which binds to RANK receptors on the surface of nearby monocytes. RANKL induces those monocytes to fuse to form a multinucleated OC cell [97, 98]. Furthermore, to keep bone resorption under control, the OBs also secrete “osteoprotegerin (OPG),” which binds to RANKL, prevents RANK receptors, and ultimately slows down the activation of OCs [99]. Osteoprogenitor, preosteoblast, and OB are the three separate steps of increased differentiation in OBs toward the skeletal lineage. With the assistance of osteoprogenitor cells, the transcription factor SOX-9 is first expressed and regulates the differentiation of chondrocytes based on their cell fate [100]. The sole cell type present in healthy cartilage is the chondrocyte, which creates a cartilaginous matrix made of collagen and proteoglycans. Additionally, the osteoprogenitor cell's preosteoblast has subsequently expressed the Runt-Related Transcription Factor 2 (RUNX2). On the other hand, preosteoblasts are impacted by WNT—catenin signalling throughout the development stage to encourage OBs [101, 102].

### 20.2. OCs or Bone-Resorbing Cells

OC origin depends upon a series of factors: (1) haemopoietic stem cell of bone marrow, (2) M-CSF which activates monocytes (a myeloid cell), (3) RANKL (a member of the tumour necrosis factor [TNF]) which helps multinucleated OCs (by fusion of monocytes) to mature, and (4) OPG to form a balance between bone formation and resorption [100, 103]. The OC starts secreting proteolytic enzymes mostly cathepsin K (collagen) and MMP9, which digest the collagen protein in the organic matrix [95, 104]. This drill pits on the bone surface known as “Howship's lacunae.” OCs within the sealing zone on the bone matrix start producing hydrochloric acid, which dissolves hydroxyapatite into soluble calcium (Ca^2+^) and phosphate ions (PO_4_^3−^), and these ions get released into the bloodstream [100, 104]. Following bone resorption, the OC starts secreting an “osteoid” seam (a substance mainly made up of collagen) to fill in the lacunae, created by OCs. Calcium and phosphate begin to deposit on the seam, forming hydroxyapatite. Also, as OBs keep producing new bony material, many get trapped within tiny lacunae within the bony matrix and turn into osteocytes [90, 105]. Bone remodelling is affected by various hormones, that is, the parathyroid glands. The parathyroid hormone travels to the bones and stimulates the OB to release RANKL which triggers bone resorption [106, 107]. This allows calcium ions (Ca^2+^) to be released into the bloodstream, and that corrects the deficiency. Now, when the blood calcium level is higher than normal, the parathyroid gland releases less parathyroid hormone to have less bone resorption [108]. In addition, “parafollicular cells” in the thyroid gland produce a hormone called “calcitonin.” High calcitonin levels inhibit bone resorption which results in lower blood calcium levels [109, 110]. Another factor in bone remodelling is mechanical stress. That is why bones that bear a lot of weight remodel at such a high rate—a phenomenon called “Wolff's law” [111]. Vitamin D also stimulates intestinal absorption of calcium, which then causes calcium levels to increase and inhibits bone resorption [112, 113]. General factors that increase the risk of osteoporosis and fractures are given in Table 2.

## 21. BC and Osteoporosis

**Figure 2:**
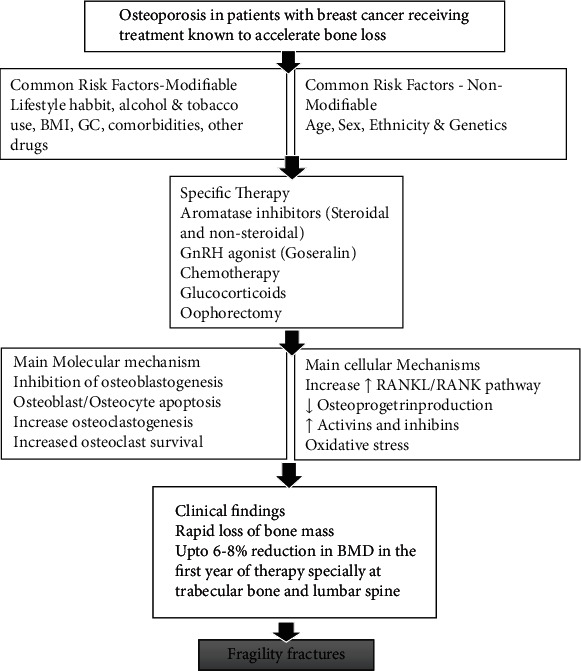
Pictorial representation of the generation of osteoporosis in patients with breast cancer receiving different treatments.

The primary causes of osteoporosis in BC involve estrogen deprivation brought on by CT and hormone treatment (HT), and more particularly, the use of nonsteroidal aromatase inhibitors (AIs) [116]. By attaching to ERs alpha and beta (ER and ER, respectively), which are expressed in both OBs and OCs, estrogens play a crucial role in maintaining the health of bones by reducing bone resorption and bone loss [117, 118]. By increasing the synthesis of OPG and decreasing the synthesis of OC differentiation and proliferation factor RANKL, estrogens increase the proliferation and activity of OBs, decrease the apoptosis of osteocytes (involved in bone formation), and reduce the differentiation and maturation of osteoclastic precursors [119–121]. Rapid bone loss, especially in trabecular bone, can result from CT (especially CT involving alkylating agents and/or 5-fluorouracil) or following HT (especially HT using nonsteroidal AIs). There is a great cellular link between osteoporosis caused by BC treatment, especially CTs. BC CTCs enter the bone via the blood arteries that nourish the bone marrow [24, 122–124]. CTCs undergo a mesenchymal-to-epithelial transition (MET) and begin producing parathyroid hormone-related peptide (PTHrP), at which point they bind to specialized stromal cells lining the bone facing the marrow [125]. PTHrP stimulates the expression of receptor activators of NF-*κ*B ligand (RANKL) and OPG in neighbouring OBs [126]. As a result, OC precursors mature into functional OCs that perform osteolysis, demineralizing the bone and exposing its extracellular matrix. During this action, transforming growth factor, calcium, bone morphogenetic proteins, fibroblast growth factors, and insulin-like growth factor-1 (IGF-1) are all produced, encouraging cancer cell proliferation and survival [127, 128]. The backbiting cycle of bone metastasis is caused by a self-sustaining positive feedback loop powered by TGF, which leads BC cells to produce more PTHrP as they multiply [129, 130]. Whereas hormone-induced ovarian failure, particularly in young women, can be restored months after withdrawal, CT-induced ovarian failure has more acute and difficult-to-reverse effects. Premenopausal women with CT-induced menopause treated with gonadotropin-releasing hormone (GnRH) agonists, women initially treated with tamoxifen (TAM) and then treated with AI, and ultimately women treated alone with AI, particularly those aged 70 years, had the highest risk of osteoporosis [131, 132].  Figure 2 is the pictorial representation of the generation of osteoporosis in patients with BC patients receiving different treatments.

## 22. From Pathophysiology and Evidence

There is a strong link between osteoporosis and BC that can be attributed to the metabolism of estrogen [133, 134]. It was established in the 1980s that, in addition to a family predisposition, the traditional risk factors for BC (early menarche, late menopause, obesity, nulliparity, and advanced age at first childbirth) are associated with an increased endogenous or exogenous estrogen exposure duration. The risk variables linked to estrogen exposure should be protective against low bone density and the onset of osteoporosis, which, on the other hand, supports the development of BC [135]. Key TJ and group in 2011 proved that circulating higher concentrations of estrogens and androgens (sex hormones) after menopause may function as a mediator to explain how these factors affect BC risk. The potential of estrogen to sustain human bone cells when administered directly through an ER was well established in the 1990s [136]. OBs and OCs are direct receptor-transmitters for the effects of estrogens on bone metabolism. Leptin, neuropeptide Y, TNF, IGF-1, interleukin (IL)-1, and IL-6 are all cytokines and mediators that have a role in estrogen's indirect effects on bone metabolism [137–139]. Because they have a proliferative effect on breast tissue, these mediators and cytokines are regarded as risk factors for the development of BC and its therapy. On the other hand, bone density and bone quality are two key characteristics that are integrated to form bone strength. BMD *T*-scores, which represent a measured value 2.5 standard deviations below the average of a control group of young women and men, are also used to characterize osteoporosis [134]. BC survivors and their doctors are increasingly concerned about osteoporosis and osteopenia, which are frequently linked to therapy. Several BC treatments, including AIs, chemotherapy-induced ovarian failure (CIOF), and antagonists or agonists of the GnRH, cause bone loss in certain women that are significant enough to lead to osteoporosis and fractures [139]. The predicted 25 million cancer survivors in the United States by 2040, most of whom will be in their sixth, seventh, and eighth decades, are very relevant to osteoporosis [140]. The presence of cancer and several oncology treatments (glucocorticoids, hormone therapy, radiotherapy, and CT) all pose distinct risks for the onset of osteoporosis, bone loss, and fractures [140, 141].

## 23. Menopause or Postmenopausal Incidences

There is a thorough balance between osteoblastic bone synthesis and osteoclastic bone resorption in the healthy reproductive state. After menopause, there is a considerable change in this process. In addition to the classic symptoms of hot flashes and mucosal shrinkage brought on by physiologically reduced plasma levels of estriol, bone health suffers severely. Despite individual differences, healthy postmenopausal women lose 1% of their bone mass per year [85].

## 24. Treatments in Postmenopausal Women With BC

In women with BC, CT and endocrine treatments play a major role in intensifying osteoporosis, as they are well known to harm bone metabolism.

### 24.1. CT

In BC patients, bone density is negatively impacted by new chemotherapeutic drugs, and bone remodelling is negatively impacted by AIs, which can reduce plasma estradiol, estrone, and estrone sulphate concentrations by up to 98% [142–144]. Increased OC activation, increased OC survival and recruitment, increased osteoblastic apoptosis, and an increase in the depth and quantity of bone remodelling units are all consequences of estrogen deprivation [145, 146] (Table 3).

Chemotherapeutic drugs including methotrexate, doxorubicin, taxane, cyclophosphamide, 5-fluorouracil, and cisplatin have been shown to have an inhibitory effect on bone formation and promote bone resorption without increasing bone metastases both in vitro and in animal models [150–152]. Additionally, glucocorticoids such as prednisone and dexamethasone increase bone resorption, decrease osteoblastic activity, alter muscular strength, alter calcium absorption and excretion, and disrupt the pathways for growth hormone and growth factor production [153]. Regardless of age or menopausal state, long-term glucocorticoid (> 7.5–15 mg of prednisone per day for > 5 years) use may lead to a 30% increase in osteoporotic fracture [154]. In animal models, the trabecular bone structure was reduced by 60%. These side effects of CT are in addition to the elevated OC activity seen in BC patients' bones [155, 156]. In case-control research involving 352 postmenopausal women, it was found that women with primary BC had a 2.8-fold higher relative risk and a fivefold higher incidence of fractures annually. Even at the start of the research, women with recurrent BC had a sixfold higher fracture incidence, a 23-fold higher incidence yearly, and a 24.5% higher relative risk [157].

## 25. Adjuvant CT

Even in the absence of clinically obvious disease, adjuvant systemic CT is frequently utilised in women with early BC after their original tumour has been treated with surgery and radiation. Unfortunately, premenopausal women who receive adjuvant CT run the danger of suffering significant bone loss and premature ovarian failure [158]. Because premenopausal women who get adjuvant CT but do not go through menopause do not experience a significant drop in BMD, it is possible that the estrogen shortage in these women is mostly to blame for bone loss [159, 160]. However, CT can directly damage bones. The fact that 75% of BC patients are postmenopausal at the time of diagnosis makes it crucial to determine if adjuvant CT poses a threat to bone health in addition to the one already posed by estrogen deprivation.

## 26. AIs

AIs reduce estrogen levels by preventing the aromatase enzyme, which is found in adipose tissue, from converting other hormones into estrogen [161]. These medications do not prevent estrogen production by the ovaries [162]. The cytochrome P450 enzyme aromatase (CYP19), which is found in a variety of tissues including the brain, fat, bone, healthy breast, and BC, transforms androgens from the adrenal gland into estrogens in postmenopausal women [163, 164]. When aromatase is inhibited, estrogen levels fall even further than the already abnormally low levels experienced by postmenopausal women [165]. The three selective AIs used to treat postmenopausal women with breast malignancies expressing the ER and/or PR are anastrozole, exemestane, and letrozole. Women receiving AIs experience a lumbar spine BMD loss of 2%–3% [147]. Randomized studies have shown that women with BC taking AIs have higher fracture rates than those taking TAM [150], and recent retrospective research has found that women taking AIs and bisphosphonates had decreased fracture risks [141]. The fracture risk remains high for the first 5 years of AI treatment and then declines at a rate comparable to that of women taking TAM from Years 5 through 10 [148].

## 27. Endocrine Treatment

It is generally established that TAM therapy increases BMD in postmenopausal women with BC. An increase in BMD was seen in women who took TAM compared to a yearly decline in the control group (0.6% vs. 1.0%, respectively), according to a randomized placebo-controlled double-blinded experiment. In the same study, women with BC who had TAM treatment had a significantly lower incidence of fractures [166, 167]. Along with the higher BMD, a notable improvement in bone structure was also seen. Regarding the mode of action, AIs work by preventing the activity of the aromatase enzyme, which lowers the level of estradiol in the blood. A decrease in BMD, an increased risk of fracture, and higher markers of bone resorption are all effects of AI-induced estrogen deprivation. Regardless of the kind of AI used, aromatase inhibitor-associated bone loss (AIBL) occurs at a rate that is at least twofold higher than the loss of BMD reported in healthy, age-matched postmenopausal women, leading to a significantly increased incidence of fractures [141, 149]. After 2 years of anastrozole treatment, bone structure as measured by the trabecular bone score (TBS) also considerably declined at the lumbar spine and the hip [168].

## 28. GnRH Antagonist, Oophorectomy, and CIOF

A GnRH antagonist is a compound that prevents the pituitary gland from producing the hormones, namely, luteinizing hormone (LH) and follicle-stimulating hormone (FSH). This stops the testicles in men from producing testosterone [169]. Treatment with a GnRH agonist (or antagonist) causes bone loss, “chemical menopause,” and is sometimes paired with TAM or AI [170]. Adjuvant CT causes primary ovarian failure, which accelerates bone loss that starts as soon as 6 months after treatment begins and continues to accelerate after 12 months [171]. In the lumbar spine, the extent of bone loss caused by GnRH agonists and CIOF is 6%–8% [172]. While degarelix primarily functions as an antagonist, leuprolide, goserelin, triptorelin, and histrelin are all regarded as GnRH agonists [173]. Because of the reduced ovarian reserve brought on by a decline in the quantity and quality of follicles, the risk of CIOF rises with age. The risk of CIOF is highest with alkylating drugs, such as cyclophosphamide, and lowest with platinum agents, anthracyclines, and taxanes. Increased risk of CIOF is associated with higher cyclophosphamide cumulative doses [174].

## 29. Selective Estrogen Receptor Modulators (SERMs)

One of three types of HT drugs called SERMs is used to treat ER-positive BC in both men and women, including those who are pre- and postmenopausal. SERMs prevent your body's natural estrogen from interacting with BC cells [175]. SERMs include TAM and raloxifene. Selective ER modulators bind to the ER, acting as an estrogen agonist or antagonist depending on the target tissue. TAM is an estrogen agonist in the bone in preclinical models [176]. TAM, however, prevents bone loss in postmenopausal women while it slightly increases bone loss in premenopausal women [14]. Based on the observation that women on TAM do not experience a reduction in the frequency of fragility fractures, the drug's bone-protective characteristics are rather ineffective [177]. Raloxifene, a medication for osteoporosis and BC prevention approved by the US FDA, raises BMD and lowers spine fractures but does not affect nonspinal fractures [178]. There are stronger and more efficient medications available to treat osteoporosis in women [179].

## 30. Other Factors for the Induction of Osteoporosis

### 30.1. Menopause and Aging

Beginning in middle age, bone loss is frequently caused solely by aging and occurs at a rate of 0.5%–1% per year in both men and women [180]. Women lose bone at a rate of 3%–5% per year for the first 5 years after menopause, which is caused mostly by a decrease in the amount of circulating estrogens. Osteoporosis affects one in every three postmenopausal women worldwide, according to estimates [181–183]. Geriatric illnesses such as a predisposition to falls, delirium, dementia, and incontinence can predispose both to osteoporotic fractures, in addition to increasing morbidity and mortality and delaying the time it takes for fractures to heal [184].

### 30.2. Vitamin D Deficiency

Other than menopause and age, secondary causes of bone loss include disease conditions that might induce osteoporosis [185]. These diseases, if present, have the potential to exacerbate the detrimental effects of BC treatment on bone health [14]. The most frequent secondary cause identified in both groups in this study was a vitamin D deficiency. These results are in line with those of other researchers who have discovered that vitamin D deficiency may be the primary cause of speeding up bone loss in people on an AI [186].

### 30.3. Idiopathic Hypercalciuria and Primary Hyperparathyroidism

The BC group exhibited a slightly greater prevalence of idiopathic hypercalciuria than the non-BC group. This disease is characterized by elevated urine calcium excretion in the absence of the use of diuretics or other disorders that cause hypercalcemia, such as high calcium or vitamin D consumption, primary hyperparathyroidism, sarcoidosis, or malignancies. Hypercalcemia affects up to 4% of the population and has been associated with cancer, primary hyperparathyroidism, eating too much calcium or vitamin D, ectopic production of 1,25-dihydroxy vitamin D (1,25(OH)2D), and inadequate 1,25(OH)2D degradation. Excessive vitamin D3 (or D2) consumption can result in hypercalcemia and hypercalciuria [187]. In healthy people, blood calcium, phosphorus, and 25 OHD levels are normally normal; however, as calcium consumption declines, serum calcium levels fall, which may increase PTH levels. Reduced PTH levels produce an increase in sclerostin, Cyp27b1, and S 1,25(OH)2D levels, which appears to promote ductal tubule calcium reabsorption and cause bone resorption or bone demineralization, resulting in osteopenia and osteoporosis [188]. Secondary causes of bone loss affect between 30% and 50% of women with osteoporosis. Vitamin D deficiency, idiopathic hypercalciuria, glucocorticoid excess, primary and secondary hyperparathyroidism, hyperthyroidism, hypogonadism, drugs, malabsorption, medication side effects, gastrointestinal disorders, hematologic disorders, Cushing's syndrome, solid organ (lung, liver, heart, and kidney) failure, and transplantation are some of the more prevalent causes of secondary osteoporosis [189, 190].

## 31. Management of Osteoporosis

**Figure 3:**
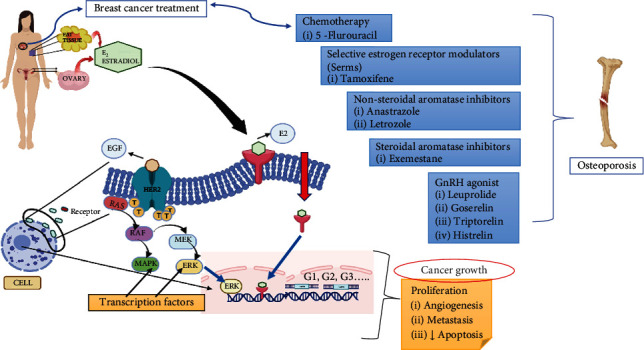
Image showing molecular and genetic level transitions in breast cancer patients that may cause osteoporosis.

Regarding osteoporosis, adequate calcium intake (1000 mg/day) and additional vitamin D (1000–2000 units/day) administration to postmenopausal women with osteopenia to prevent bone loss are well-proven [191]. Calcium intake over a lifetime has been shown to reduce the risk of osteoporosis by up to 20%. A review of 45 studies involving patients in nursing homes found that those who take calcium and vitamin D supplements have a lower likelihood of hip fractures [192]. A meta-analysis of postmenopausal-aged women and men (*n* = 45,509) revealed a lower fracture risk of 18%, which supported this finding [192]. Administration of calcium and vitamin D, however, is insufficient to stop AIBL. Apart from supplements (calcium and vitamin D) intake, certain lifestyle changes are mandatory for curing osteoporosis. Amongst all the management procedures for osteoporosis, bisphosphonates grab the first position as a first-line treatment for curing bone fragility issues. Alendronate, zoledronate, risedronate, and ibandronate are some of the bisphosphonates which are drugs used to treat fragile bones [193]. However, every treatment has its sort of side effects, and one of them is situated with bisphosphonates. When taking bisphosphonates orally with a glass full of water, a person should sit in an upright position, not lying on the bed, as it causes severe ulcers throughout the oesophagus and stomach [194, 195]. Similarly, with denosumab treatment, skin eczema, flatulence, cellulitis, and osteonecrosis of the jaw were seen [196]. TAM and raloxifene also have some severe adverse reactions. TAM depicts some severe pharmacodynamic effects, and a noncardioprotective drug causes thromboembolism and fatty liver. It also increases the oxygen species pathway within mitochondria, leading to cell apoptosis and induced aging [197]. Likewise, raloxifene leads to blood clots and increases the risk of death due to stroke [198].  Figure 3 demonstrates the complex interaction between estrogen signalling and BC development, focusing on how estradiol (E2), produced by adipose tissue, is a key driver of cancer cell proliferation. Estradiol binds to its receptors, initiating the activation of transcription factors such as ERK, which regulates cell cycle progression (G1, G2, and G3 phases) and contributes to tumour growth by enhancing processes like proliferation, angiogenesis, metastasis, and the reduction of apoptosis.

To counteract this, BC therapies often target the estrogen pathway. These treatments include CT agents like 5-fluorouracil, SERMs such as TAM, and AIs (both nonsteroidal like anastrozole and letrozole and steroidal like exemestane). Additionally, GnRH agonists such as leuprolide and goserelin are employed to decrease estrogen production. However, one significant side effect of these estrogen-blocking treatments is an increased risk of osteoporosis, as estrogen is essential for bone health.  Figure 3 effectively illustrates the connection between these therapies and their side effects, underscoring the challenge of balancing cancer treatment with the preservation of bone integrity.

## 32. Discussion

BC treatment can cause osteoporosis. At the same time, studies have reported that most of the anticancerous drugs used in the treatment of BC are well known to produce osteoporotic effects. In 2012, Hadji testified that several generations of SERMs have been developed for the prevention and treatment of postmenopausal osteoporosis. Third-generation SERMs (lasofoxifene, bazedoxifene, and raloxifene) exhibit ER agonist and antagonist activity depending on the target tissue and thereby improve bone health. According to the study, it is stated that a perfect SERM would shield bone without triggering breast or endometrial activity and only bazedoxifene reaches clinical use. Apart from that, first-generation SERMs (TAM), which is a first-line treatment for ER-positive BC, depict ER antagonistic activity in the breast and endometrial. Osteoporosis in cancer patients has now become a common phenomenon. This is due to anticancer drugs that, in some cases, when taken for a longer period, can cause bone-related diseases, and one of them is osteoporosis. All of these lead to therapy that involves the simultaneous administration of anticancer and antiosteoporotic drugs. Since osteoporosis is a progressive disease, cumulative targeted approaches in the early stage of disease development are necessary.

## 33. Conclusion

The text highlights the current state of research on BC diagnosis and treatment, emphasizing the need for further research on early detection. Despite recent developments in techniques for detection and therapy guidance, these techniques have limitations that prevent their use as standalone tools. To plan effective treatment, it is important to understand the molecular heterogeneity of the tumour, and acute stratification is necessary to identify different groups and subgroups, each with its own unique prognosis and systemic therapy requirements. The tone is objective and professional, geared toward a scientific scholar audience. BC and osteoporosis are two significant health issues affecting a large number of people worldwide. Research has established that there is a link between these two conditions. Osteoporosis is more prevalent in females, and the decline in estrogen levels during menopause hastens the condition. Women who have had BC or are at high risk of developing it are often not advised to undergo hormone replacement therapy, which increases the risk of osteoporosis. BC treatments that reduce bone density also increase the risk of osteoporosis and fractures. Studies have suggested that CT-induced estrogen deprivation could be a possible cause of the link between BC and osteoporosis. Further research is needed to identify the underlying mechanisms and pathways of this connection.

## Figures and Tables

**Table 1:** The spread of breast cancer metastasis to different organs.

**Metastasis to organs**	**Spreadability %**	**Types of intrinsic factors involved in metastasis**	**References**
Bone	67%	Luminal A—79%	[1, 26, 39]
		Luminal B—75%	
		HER2^+^—60%	
		Basal-like—40%	

Liver	40.8%	Basal-like (TNBC) and HER2^+^ are more frequent than others	[1, 25, 39]

Lung	36.9%	Luminal A—25%	[32, 34, 39]
		Luminal B—30%	
		HER2^+^—45%	
		Basal-like—35%	

Brain	12.6%	Luminal A—< 10%	[25, 26, 40]
		Luminal B—10%	
		HER2^+^—30%	
		Basal-like—25%	

Auxillary lymph nodes	30%–50%	Not specified	[39, 40]
Mamary internal chain lymph nodes	10%–40%		
Supraclavicular lymph nodes	1%–4%		
Contralateral breast	6%		

**Table 2:** General factors that increase the risk of osteoporosis and fractures [114, 115].

**Primary factors of osteoporosis**	
*Nonmodifiable risk factors*	
*Factors*	*Possible cause/risk*
Age	Personal history of the previous fracture
Gender	Genetic (family history)
Ethnicity (Asian and Caucasian)	Caucasian and Asian women are at highest risk
*Modifiable risk factors*	
*Factors*	*Possible cause/risk*
Low levels of physical activity (prolonged immobilization and/or sedentary lifestyle)	Estrogen deficiency (early menopause, prolonged amenorrhea periods)
Smoking	Low calcium intake or malnutrition
Alcohol consumption (≥ 3 units/day)	Osteoporosis secondary to chronic or consumptive diseases
Low weight (< 58 kg or 127 lb)	Chronic glucocorticoid use
*Drugs used in oncology*	
Aromatase inhibitors (BC)	Chemotherapy
Steroidal (exemestane)	Alkylating agents
Nonsteroidal (anastrozole and letrozole)	Anthracyclines and docetaxel
GnRH agonists (BC: goserelin and triptorelin)	Doxorubicin and excessive alcohol consumption
Selective ER modulators (BC)	5-Fluorouracil
LHRH analogues (goserelin, buserelin, leuprorelin, and triptorelin)	Antidepressants and serotonin reuptake inhibitors
LHRH antagonists (goserelin)	Oral antidiabetics (thiazolidinediones)
Antiandrogens (enzalutamide, bicalutamide, flutamide, and nilutamide)	
*Other osteopenizing drugs*	
Methotrexate	NSAID category drugs
Megestrol acetate	Estramustine
Platinum compounds	Ifosfamide
Cyclophosphamide	Radiotherapy and hypogonadism
Interferon alfa	Combination of chemotherapy regimens
Cyclosporine	Valproic acid

**Secondary factors of osteoporosis**	
Amenorrhoea	Malabsorption
Long-term or high-dose oral corticosteroid use	Smoking
Hyperthyroidism	Female hypogonadism
Anorexia nervosa	Male hypogonadism
Malignant disease	Hyperprolactinaemia
Multiple sclerosis/chronic inflammatory arthritis	Cushing's syndrome
Immobilization	Thyroxine treatment
Rheumatoid arthritis	Chronic inflammatory bowel disease
Myeloma	Excessive alcohol intake
Treatments for endometriosis	Chronic liver disease

Abbreviations: BC, breast cancer; ER, estrogen receptor; GnRH, gonadotropin-releasing hormone; kg, kilograms; lb pounds; LHRH, luteinizing hormone-releasing hormone; NSAIDs, nonsteroidal anti-inflammatory drugs; PC, prostate cancer.

**Table 3:** Approaches to treating bone metastases [147–149].

**Therapy**	**Mechanism**	**Stage of clinical development**
Bisphosphonates	Block bone resorption; might block tumour-cell mitosis and stimulate tumour-cell apoptosis; alleviate bone pain	On the market
Denosumab	A human antibody that is effective in preventing bone loss and bone deterioration	On the market
Targeted therapy		
(a) Monoclonal antibodies (palbocilib, ribociclib, and abemaciclib)	Trigger an immune system response that can destroy the outer wall of a cancer cell	On the market
(b) With HER2 negative breast cancer (olaparib and talazoparib)	Trap the PARP-1 protein at a single-stranded break and disrupt its catalytic cycle, leading to replication fork progression and double-strand breaks	Phase III
(c) With metastatic breast cancer (trastuzumab deruxtecan)	Inhibits HER2 homodimerization, thereby preventing HER2-mediated signalling	Phase III
Osteoprotegerin	Prevents RANKL from binding its receptor and stimulating osteoclasts	Phase II
RANK-Fc	Prevents RANKL from binding its receptor and stimulating osteoclasts	Phase I
PTHrP antibodies	Neutralize PTHrP	Phase III
Vitamin D analogues	Decrease PTHrP production	Phase III
Hormonal therapy (tamoxifen, anastrozole, exemestane, letrozole, gasorelin, fulvestrant, and elacestrant)	Restore the balance between bone resorption and formation, slowing bone loss and increasing bone mass	On the market (generic)

Abbreviations: PTHrP, parathyroid-hormone-related peptide; RANK, receptor activator of nuclear factor-*κ*B; RANKL, receptor activator of nuclear factor-*κ*B ligand.

## Data Availability

The data will be made available on request.
